# Neoadjuvant Treatment in Pancreatic Cancer

**DOI:** 10.3389/fonc.2020.00245

**Published:** 2020-02-28

**Authors:** Atsushi Oba, Felix Ho, Quoc Riccardo Bao, Mohammed H. Al-Musawi, Richard D. Schulick, Marco Del Chiaro

**Affiliations:** ^1^Division of Surgical Oncology, Department of Surgery, University of Colorado, Anschutz Medical Campus, Denver, CO, United States; ^2^Department of Hepatobiliary and Pancreatic Surgery, Cancer Institute Hospital, Japanese Foundation for Cancer Research, Tokyo, Japan; ^3^Department of Surgery, Oncology, and Gastroenterology, University of Padua, Padua, Italy; ^4^Clinical Trials Office, Department of Surgery, University of Colorado, Anschutz Medical Campus, Denver, CO, United States

**Keywords:** neoadjuvant therapy, neoadjuvant chemotherapy, neoadjuvant chemoradiotherapy, borderline resectable, locally advanced, FOLFIRINOX, gemcitabine, nab-paclitaxel

## Abstract

Thanks to the development of modern chemotherapeutic regimens, survival after surgery for pancreatic ductal adenocarcinoma (PDAC) has improved and pancreatologists worldwide agree that the treatment of PDAC demands a multidisciplinary approach. Neoadjuvant treatment (NAT) plays a major role in the treatment of PDAC since only about 20% of patients are considered resectable at the time of diagnosis. Moreover, increasing data demonstrating the benefits of NAT for borderline resectable/locally advanced PDAC are driving a shift from up-front surgery to NAT in the multidisciplinary treatment of even resectable PDAC. Our understanding of the role of NAT in PDAC has evolved from tumor shrinkage to controlling potential micrometastases and selecting patients who may benefit from radical resection. The present review gives an overview on the current literature of NAT concepts for BR/LA PDAC and resectable PDAC.

## Introduction

The role of neoadjuvant treatment (NAT) in pancreatic adenocarcinoma (PDAC) is still under debate due to a relative lack of robust data compared with other gastrointestinal cancers, in which the role of NAT is more well-defined. For example, in esophageal cancer neoadjuvant chemoradiotherapy is standard of care for resectable disease, and is associated with improved OS, DFS, pathological Complete Response (pCR), and R0 resection rate as shown in phase III RCT (1–3). The survival benefit of neoadjuvant chemotherapy has also been reported in phase III RCT in resectable gastric cancer (4–6). Moreover, neoadjuvant chemoradiotherapy has become widely accepted as standard of care for resectable rectal cancer in the last decades, as up to 50–60% of patients are downstaged after neoadjuvant chemoradiotherapy (7–9), and up to 25% of the patients have a pCR (10). While these encouraging data have led to the development of new therapeutic approaches, permitting even organ-sparing treatments for rectal cancer (11), this has not carried over to PDAC. Several barriers have limited the application of NAT in pancreatic cancer. First, historically, the response rate to chemotherapy for pancreatic cancer was very low (12). The persistence of this perspective has contributed to low patient compliance in accepting NAT in clinical trials, particularly earlier ones (13). This has been mitigated somewhat by improved chemotherapy regimens. Another barrier is the inability of current radiological modalities to adequately define the level of response to NAT, because restaging after NAT is based on imaging findings on CT and MRI scan, which are not predictive of resectability or pathological response (14, 15). Furthermore, retrospective cohort studies report a lower pCR rate for PDAC than other GI malignancies, ranging between 2 and 15% (16–18). Median OS in patients who do attain pCR appears to be longer, but it is difficult to assess the impact on survival of pCR with such low pCR rates.

According to 2019 NCCN guidelines, NAT is now the accepted approach for borderline resectable (BR) disease, while upfront surgery is still the recommendation for resectable disease except in cases with high risk features (19). Since only about 20% of patients are considered resectable at diagnosis (20), the use of NAT plays a major role in the treatment of PDAC. The preferred regimens in the neoadjuvant/adjuvant setting, the first-line therapy for locally advanced (LA) and metastatic disease, are FOLFIRINOX (or modified FOLFIRINOX) or gemcitabine + nab-paclitaxel (GnP). In 2003, a French group first reported on the feasibility of FOLFIRINOX in metastatic solid tumor in a phase I trial, including two metastatic PDAC patients (21). Since then, phase II RCTs have studied the effects of FOLFIRINOX in metastatic PDAC, showing a response rate >30% (22, 23). In the phase III PRODIGE RCT, patients with metastatic PDAC treated with FOLFIRINOX were reported to have a 11 months median OS, vs. 6.8 months in gemcitabine group, and a PFS 6.4 vs. 3.3 months, respectively (24). In 2011, phase I/II trial results with 67 patients with advanced PDAC treated with GnP were published, showing a 12.2 months OS, and 48% partial response rate (25). Based on these results, the phase III MPACT trial compared GnP vs. gemcitabine alone in 861 metastatic PDAC patients. Primary endpoint was reached, showing a 8.7 vs. 6.6 months OS (26), and GnP group was also associated with a improved 1- and 2-year survival, response rate and PFS (27). These encouraging data reported in recent years has led to an increasing number of patients treated with NAT using FOLFIRINOX and GnP regimens, even in resectable disease.

Another important advantage of treatment with NAT is an increase in the proportion of patients who receive chemotherapy. This is based on the rationale that adjuvant chemotherapy (AC) has been shown to improve survival in phase III RCTs. The CONKO-001 trial randomized 369 patients without prior chemotherapy into gemcitabine AC group vs. observation groups. This study showed a statistically significant difference in survival (median OS 22.8 vs. 20.2 months, respectively, median DFS 13.4 vs. 6.9 months) (28). More recently, the PRODIGE-24 trial randomized 493 patients to receive mFOLFIRINOX or gemcitabine in adjuvant setting. The mFOLFIRINOX regimen showed a longer survival than gemcitabine (median OS was 54.4 vs. 35.0 months, median DFS 21.6 vs. 12.8 months, respectively) (29). Traditionally, only patients with a good performance status and a good recovery after surgery are treated with AC. About 45% of patients do not receive AC after resection due to poor performance status, postoperative morbidity, or early progression of disease (30, 31). This may lead to a decreased survival in these patients. Given the large amount of data showing the survival benefit of chemotherapy both in the neoadjuvant and adjuvant setting when compared with no chemotherapy, it has become clear that chemotherapy, either before or after surgery, is a crucial component in the treatment in PDAC.

## Neoadjuvant Therapy in Borderline Resectable and Locally Advanced PDAC

The purpose of NAT in BR/LA PDAC is not necessarily to decrease tumor size to facilitate an easier resection, but to select those candidates for radical resection who do not have tumor progression that would indicate biologically-aggressive disease. Indeed, several papers have shown favorable outcomes after resections following NAT, despite post-NAT imaging suggesting persistent unresectability (14, 15). A few specialized centers have even reported favorable long term survival in patients who undergo pancreatectomy with concomitant arterial resection and reconstruction following NAT (32, 33). Tee et al. reported 2-year OS of 62.3% in 65 patients who underwent pancreatectomy with arterial resection following NAT, which was superior to upfront resection (25.8%, *p* = 0.038, log-rank test) (32). Del Chiaro et al. reported 5-year survival of 23.4% in 34 patients who underwent pancreatectomy with arterial resection (half of whom had undergone NAT) compared with 0% in 39 patients with BR/LA disease who underwent exploration with curative intent but ultimately were treated palliatively due to technical unresectability (0%, *P* = 0.003). The surgical complication rate was feasible at 38.2% and mortality rate was low at 2.9% (33). These favorable results can be attributed not only to improved surgical skills and perioperative management, but also to modern chemotherapeutics controlling potential micrometastases and selecting patients who may benefit from radical resection after NAT (33).

Surgical resection for LA disease following NAT continues to be debated. Michelakos et al. analyzed 110 resected BR/LA patients after FOLFIRINOX, and in the absence of reliable predictors of resectability advocated that all BR/LA patients with no progression on NAT should be offered surgical exploration (34). Similarly, Rangelova et al. analyzed 154 resected BR/LA patients after NAT and suggested that every patient who receives NAT for BR/LA PDAC without radiological signs of disease progression should undergo exploration with intent of resection because it is not possible radiologically to define regression criteria (35). Moreover, they showed that surgical resection had a positive impact on survival for all values of CA 19-9 despite the fact that higher levels of CA 19-9 have been associated with worse prognosis (35). On the other hand, Satoi et al. describe a relatively high early recurrence rate of 30% within 6 months after surgical resection for LA disease following NAT, highlighting a need for more judicious use of surgery in this setting. The decision process should include a multidisciplinary discussion and consideration of radiologic findings (e.g., reassuring findings include stable disease or partial response) as well as CA 19-9 levels (e.g., decreased CA 19-9 <100 U/ml) (34, 36, 37).

One main marker of effectiveness of NAT in BR/LA patients is the proportion of patients who proceed to resection, but the best regimen for BR/LA patients is still controversial. Based on the results from RCTs in metastatic patients, FOLFIRINOX and GnP are currently considered the two best chemotherapy regimens for BR/LA patients. The Heidelberg group for example reported 125 patients with locally advanced PDAC treated by FOLFIRINOX in NA setting. Resection rate was 61% and the median OS after resection was 16.0 months, and FOLFIRINOX was confirmed to be independently associated with a favorable prognosis (38). More recently, the Karolinska group reported on 156 patients treated with NAT for BR/LA PDAC, including 34.6% with FOLFIRINOX and 15.4% with GnP. Exploration was attempted in 76 patients (48.7%), and resection was performed in 52 patients. Median survival after resection was 22.4 vs. 12.7 months in non-resected group. Interestingly, while dose reductions of other regimens were associated with impaired OS, dose reduction in FOLFIRINOX did not impact overall survival (35). Macedo et al. compared resected BR/LA patients who received FOLFIRINOX vs. GnP retrospectively and revealed there was no difference between the two regimens for median local recurrence-free survival (FOLFIRINOX 23.7 months vs. GnP 17.8 months), median metastasis-free survival (23 vs. 21.2 months), overall survival (37.3 vs. 31.9 months), R0 resection rate (82.8 vs. 81.8%), ypN0 (48.9 vs. 45.6%), and normalization of CA19-9 after NAT (35.9 vs. 35.2%) (18).

FOLFIRINOX is the most commonly used chemotherapy, but there are many reports of using radiation therapy concurrently or following chemotherapy for BR/LA patients. The resection rate of FOLFIRINOX with radiotherapy for BR/LA patients has been reported to be 58–85% for BR and 13–44% for LA (39–50). compared with resection rates 51–100% for BR, 13–61% for LA when treated with FOLFIRINOX without radiotherapy (35, 38, 51–57). Although the addition of radiotherapy does not appear to make a significant difference in resectability rates and survival (refer to tables), these results are primarily from retrospective studies and may be biased, as patients who received radiation may have had more advanced disease.

Regarding GnP, there are fewer reports than with FOLFIRINOX (58–62). As many papers on GnP combined with other chemotherapy or radiation therapy, it may be considered difficult to convert LA to resectable by GnP alone (58, 59, 61, 62). In the largest phase II study (LAPACT), 107 LA patients received GnP alone and the resection rate was only 15% and R0 resection rate was 44% (60).

Other treatments are summarized in Table 1. There are many variations of regimens based on gemcitabine, with resection rates ranging from 48 to 86% for BR and 4–89% for LA, respectively (63–73).

**Table 1:** Neoadjuvant therapy in BR/LA PDAC.

**References**	**Type of study**	**Treatment**	**Total no. of patients**	**Resectability**	**No. of patients**		**Resection rate (%)**		**R0 (%)**		**mOS (mo)**	
					**BR**	**LA**	**BR**	**LA**	**BR**	**LA**	**BR**	**LA**
**FOLFIRINOX** **+** **RTx**												
Hosein et al. (39)	Retrospective	FOLFIRINOX ± CRTx	18	BR/LA	18		50		88		x	
Boone et al. (40)	Retrospective	FOLFIRINOX ± RTx	25	BR/LA	12	13	58	15	85	50	x	x
Faris et al. (41)	Retrospective	FOLFIRINOX + CRTx	22	LA		22		23		100		x
Christians et al. (42)	Retrospective	FOLFIRINOX + CRTx	18	BR	18		67		100		22	
Paniccia et al. (43)	Retrospective	FOLFIRINOX ± CRTx	18	BR	18		85		100		x	
Marthey et al. (46)	Retrospective	FOLFIRINOX ± RTx	77	LA/M		77		36		89		22
Sadot et al. (48)	Retrospective	FOLFIRINOX ± CRTx	101	LA		101		31		55		25
Blazer et al. (44)	Retrospective	FOLFIRINOX ± CRTx	43	BR/LA	18	25	61	44	82	91	21.2	
Khushman et al. (45)	Retrospective	FOLFIRINOX ± CRTx	51	BR/LA	11	40	22	91	35			
Nanda et al. (47)	Retrospective	FOLFIRINOX + CRTx	29	BR/LA	14	15	83	13	83	19		
Suker et al. (50)	Meta-analysis	FOLFIRINOX ± CRTx	315	LA		315		26		74		24
Katz et al. (49)	Phase II	FOLFIRINOX + CRTx	22	BR	22		68		93		22	
**FOLFIRINOX**												
Peddi et al. (51)	Retrospective	FOLFIRINOX	61	BR/LA/M	19	4	100	21	x	x	x	x
Tinchon et al. (53)	Retrospective	FOLFIRINOX	12	BR	12		83		x		x	
Gunturu et al. (52)	Retrospective	FOLFIRINOX	35	LA/M		16		13		x		x
Nitsche et al. (54)	Retrospective	FOLFIRINOX	14	LA		14		29		75		x
Hackert et al. (38)	Retrospective	FOLFIRINOX	125	LA/M		64		61		41		16
Yoo et al. (55)	Phase II	FOLFIRINOX	18	BR	18		67		75		21	
Barendoim et al. (56)	Retrospective	FOLFIRINOX	53	BR/LA	23	30	87	10	100	100	28	x
Byun et al. (57)	Retrospective	FOLFIRINOX	337	BR/LA/M	67	135	51	14	79	79	35	21
Rangelova et al. (35)	Retrospective	FOLFIRINOX	154	BR/LA	22	132	33		x	x	31.9	21.8
**GnP**												
Kunzmann et al. (58)	Pilot	GnP (followed by FOLFIRINOX)	8	LA		8		37		100		x
Reni et al. (59)	Phase I	GnP/Cape/Cis	24	BR/LA	6	18	25		50		18	
Takahashi et al. (62)	Phase I	GnP + CRTx	38	BR	15		74		96		x	
Hammel et al. (60)	Phase II	GnP	107	LA		107		15		44		x
Reni et al. (61)	Phase II	GnP (±Cape/Cis)	54	BR/LA	25	29	32		44		19	
**GEMCITABINE-BASED**												
Lee et al. (63)	Phase II	Gem/Cape	43	BR/LA	18	25	61	24	82	83	17	
Katz et al. (64)	Retrospective	Gem-based + CRTx	129	BR	129		66		95		33	
Kim et al. (65)	Phase II	GemOx + RTx	68	R/BR/LA	39	6	62	17	x	x	18	9
Motoi et al. (66)	Phase II	Gem/S1	35	R/BR	16		86		87		20	
Rose et al. (67)	Retrospective	Gem/Doc	64	BR	64		48		87		22	
Sherman et al. (68)	Prospective	Gem/Doc/Cape + CRTx	45	LA		45		89		70		29
Hammel et al. (69)	Phase III	Gem ± Erlo ± CRTx	442	LA		442		4		61		13
Fiore et al. (70)	Phase II	GemOx + CRTx	34	BR/LA	7	27	55		100		22	14
Busquets et al. (71)	Retrospective	GemOx ± Erlo ± CRTx	22	BR	22		50		63		13	
Eguchi et al. (72)	Phase II	Gem/S1 + RTx	34	BR/LA	13	21	15		80		13	14
Saito et al. (73)	Phase II	Gem/S1/LV	24	BR/LA	21	3	61		93		22	

*RTx, radiotherapy; mOS (mo), median overall survival (months); BR, borderline resectable; LA, locally advanced; CRTx, chemoradiotherapy; M, metastatic disease; GnP, Gemcitabine + nab-paclitaxel; Cape, capecitabine; Cis, cisplatin; Gem, gemcitabine; CRTx, chemoradiotherapy; GemOx, gemcitabine + oxaliplatin; RTx, radiotherapy; Doc, docetaxel; Erlo, erlotinib; LV, leucovorin*.

Due to lack of evidence from RCTs, the additional value of neoadjuvant radiotherapy is still under debate. The results of the ESPAC-1 trial, which showed a significant survival benefit with adjuvant chemotherapy but negative effect with adjuvant chemoradiotherapy, have largely led European centers to minimize the use of neoadjuvant radiotherapy. However, neoadjuvant radiotherapy continues to be commonly used in the United States (74). In addition to traditional external-beam radiotherapy, other modalities of radiotherapy have been developed and utilized in this setting. Keane et al. reported that the use of intraoperative radiotherapy after NAT was well-tolerated, and associated with encouraging median survival rates when incorporated into treatment of patients with unresectable disease or close or positive margins after resection for BR PDAC (75). Furthermore, newer techniques to minimize dose to the radiosensitive tissues in the abdomen including stereotactic body radiation therapy (SBRT) and intensity-modulated radiation therapy (IMRT) are increasingly used in the neoadjuvant setting for patients with BR/LA PDAC (76). However, there are few studies evaluating the impact of these modalities on surgical resection. Well-planned clinical trials to evaluate the efficacy of modern radiation therapy for BR/LA PDAC are needed.

## Neoadjuvant Treatment in Resectable PDAC

While BR/LA PDAC is increasingly treated with neoadjuvant chemotherapy, the standard treatment for resectable PDAC currently remains upfront surgery followed by AC (77). This is based on multiple trials, including the CONKO-001, ESPAC-4, and Prodige 24 studies, which have demonstrated that adjuvant systemic therapy increases disease free survival and long term survival (29, 78, 79). In the Prodige study, a RCT comparing a modified FOLFIRINOX regimen with gemcitabine in the adjuvant setting found an impressive median survival of 54 months with mFOLFIRINOX (29). Unfortunately, there is a significant fraction of patients who undergo upfront surgery but do not recover adequate functional status to receive adjuvant therapy and its treatment benefits (80).

The success of neoadjuvant chemotherapy in BR/LA has led some to raise the question of whether administering chemotherapy before surgery might confer similar benefits for resectable disease. A similar precedent exists in other gastrointestinal malignancies. One notable example is esophageal cancer, in which the impact of neoadjuvant therapy on improved survival and conversion to resectability for locally advanced and initially unresectable disease has led to increasing application of NAT for even resectable disease (1–3). The argument in favor of neoadjuvant chemotherapy in resectable PDAC is multifaceted. One proposed benefit is that it allows for earlier treatment of micrometastatic disease, which is likely responsible for the high failure rate after surgical resection for radiologically resectable disease. Secondly, by timing chemotherapy before the physiologic stress of surgery, it may allow more patients to receive a full course of cytotoxic chemotherapy. Finally, as with BR/LA disease, it may allow for better surgical selection of patients, as more aggressive disease will “declare itself” by progressing during chemotherapy thus sparing the patient the morbidity of a surgical operation that may be of limited utility.

On the other hand, there are also several arguments against neoadjuvant therapy for resectable disease. It delays surgery, especially when patients experience significant complications such as biliary occlusion, potentially allowing the cancer to progress to a point that becomes unresectable. Another issue is that unlike surgery, the initiation of chemotherapy requires a positive biopsy. This can be elusive given the cancer's anatomic location as well as its structure—which often consists of low cellularity and high stromal content—and can thus postpone therapy (81). Endoscopic ultrasound guided biopsy has a reported specificity of 96–98% but sensitivity of only 85–92%, with repeat procedure required in up to 11% of cases (82). The cellular structure of pancreatic cancer may also have implications that limit the effectiveness of neoadjuvant chemotherapy, as it is possible that the reduction of positive resection margins after NAT may be due to reduction in the density of cancer cells rather than tumor shrinkage (83). Indeed, excluding patients with a complete pathologic response, there does not appear to be a clear correlation between histologic tumor regression and survival (84).

Multiple trials have been conducted to assess neoadjuvant chemotherapy for resectable disease (13, 85–94). Most early trials were cohort studies and involved older single agent chemotherapy regimens such as gemcitabine and 5-fluorouracil, plus or minus radiation. A summary of select studies can be found in Table 2. Heinrich et al. studied the effect of neoadjuvant gemcitabine and cisplatin in 28 patients. Ninety-three percent underwent resection, and median overall survival was 26.5 months (87). Varadhachary et al. found a higher median overall survival rate of 31 months when radiation was added to neoadjuvant gemcitabine/cisplatin in a cohort of 90 patients; however, only 58% of patients underwent resection (95). In a larger study by Takahashi et al., 87% of 188 patients with resectable disease who were treated with preoperative gemcitabine and radiation underwent resection; of these patients, 99% had a R0 resection, and 5-year overall survival was 57% (90). Other studies of gemcitabine-based neoadjuvant therapy show resection rate of 74–86% and median overall survival of 17.4–27.2 months (13, 85, 86, 89, 91).

**Table 2:** Neoadjuvant treatment in resectable PDAC.

**References**	**Type of study**	**Treatment**	**No. of patients**	**Resection rate (%)**	**R0 (%)**	**mOS (mo)**
**RESULTS OF NEOADJUVANT THERAPY FOR RESECTABLE PDAC**						
**Neoadjuvant chemotherapy alone**						
Heinrich et al. (87)	Phase II	Gem/Cis	28	89	80	27
Tajima et al. (89)	Retrospcective	Gem/S1	34	100	85	56% 2-year OS
O'Reilly et al. (91)	Phase II	GemOx	38	71	74	27
Unno et al. (94)	Phase II/III	Gem/S1	362			37
**Neoadjuvant chemoradiotherapy**						
Talamonti et al. (85)	Phase II	Gem + CRTx	22	85	80	26 (resected)
Evans et al. (86)	Phase II	Gem + CRTx	86	74	86	22
Turrini et al. (88)	Retrospective	5-FU/Cis + CRTx	102	61	92	17
Takahashi et al. (90)	Phase II	Gem + CRTx	188	97	99	57% 5-year OS (resected)
Golcher et al. (13)	Phase II	Gem/Cis + CRTx	29	66	52	17
Okano et al. (93)	Phase II	S1 + CRTx	33	97	98	83% 2-year OS
Grose et al. (92)	Retrospective	FOLFIRINOX or Gem + Cape	45	40	71	22
**Neoadjuvant chemotherapy** **+** **adjuvant chemoradiation**						
Varadhachary et al. (95)	Phase II	Gem/Cis –> Gem + RTx	90	58	96	19
**References**	**Study phase**	**Treatment**	**Number planned**	**Resectability**	**Primary outcome measures**	**ClinicalTrials.gov indentifier**
**ONGOING NEOADJUVANT TRIALS FOR RESECTABLE PDAC**						
Hozaeel et al. (96)	Phase II/III	FOLFIRINOX vs. Upfront surgery	126	R/BR	Median overall survival	NCT02172976
Labori et al. (97)	Phase III	FOLFIRINOX vs. Upfront surgery	90	R	Overall mortality	NCT02919787
Heinrich et al. (98)	Phase III	GemOx vs. Upfront surgery	155	R	Progression free survival	NCT01314027
Sohal et al. (99)	Phase II	FOLFIRINOX vs. GnP	118	R	2-year overall survival	NCT02562716

*mOS (mo), median overall survival (months); Gem, gemcitabine; Cis, cisplatin; GnP, Gemcitabine + nab-paclitaxel; GemOx, gemcitabine + oxaliplatin; CRTx, chemoradiotherapy; Cape, capecitabine; OS, overall survival; R, resectable; BR, borderline resectable*.

At least two early phase II randomized controlled trials comparing gemcitabine-based neoadjuvant chemoradiation with surgery for primary resectable cancer found NAT to be safe, feasible, and efficacious, but were terminated early due to slow enrollment and did not obtain statistically significant results due to lack of power (13, 100). After Okano et al. studied the effect of S1 (an oral fluoropyrimidine derivative) plus radiation in 57 patients, and found a 2-year survival rate of 83% (93), one prospective randomized trial in Japan comparing neoadjuvant chemotherapy using gemcitabine and S1 with upfront surgery showed significant survival benefits with NAT. In this phase III trial from January 2013 to January 2016, 362 patients were enrolled across 57 centers and randomly assigned to neoadjuvant chemotherapy using gemcitabine and S1 or upfront surgery. The median overall survival in the NAT group was 36.7 months compared with 26.6 months in the upfront surgery group (*p* = 0.015) (94).

At the recent annual ASCO meeting in 2018, van Tienhoven et al. reported preliminary findings in their trial PREOPANC-1, a Dutch prospective randomized phase III trial comparing preoperative gemcitabine-based chemoradiotherapy vs. immediate surgery in resectable and borderline resectable pancreatic adenocarcinoma (101). Key significant findings included a longer disease free interval (9.9 vs. 7.9 months) and higher median overall survival rate in the preoperative treatment group, particularly among patients in whom the tumor was removed successfully (42.1 months with preoperative treatment vs. 16.8 months with immediate surgery) (101). Of note, these preliminary results were not stratified by primary resectable vs. borderline resectable disease. Nevertheless, the findings are intriguing. There are several other ongoing prospective trials evaluating various chemotherapy regimens in the neoadjuvant setting for resectable disease, including FOLFIRINOX vs. upfront surgery (NEPAFOX, NorPACT-1), and gemcitabine + oxaliplatin vs. upfront surgery (NEOPAC) (96–98). Another group is comparing neoadjuvant mFOLFIRINOX vs. GnP (Table 2) (99). The results of these trials should soon shed more light on this issue.

## Immunotherapies and Targeted Therapies

There are currently limited data available to support the use of immunotherapy for PDAC. The immune checkpoint inhibitor trial unfortunately failed to show efficacy of anti-PD-L1 therapy in advanced PDAC patients, which has been attributed to the poor immunogenicity and immunosuppressive tumor microenvironment of pancreas cancer (102, 103). However, comprehensive genomic profiling has found deficiencies in small subsets of patients that may be targets for intervention, notably BRCA 1/2 and mismatch repair (MMR). Although incidence of BRCA 1/2 mutation or MMR deficiencies in patients with PDAC is low (7 or 1%, respectively) (104, 105), the clinical trials of novel therapies against these targets have shown some promise (106, 107). For example, MMR deficient tumors were found to be susceptible to immunotherapy across multiple solid tumors including PDAC, which led to FDA approval of pembrolizumab for patients with advanced disease that have this mutation (107). Indeed, recent treatment guidelines for the management of advanced PDAC now recommend testing for mismatch repair deficiencies despite its low prevalence, due to the potential for sustained disease remission, and recommend pembrolizumab as a second line treatment in patients who test positive for MMR deficiency (108). Meanwhile, the POLO phase III trial showed the efficacy of olaparib, a PARP inhibitor, as maintenance therapy in patients who had a germline BRCA1/2 mutation (PFS; 7.4 vs. 3.8 months with placebo, *p* = 0.004) (109). As a result, olaparib is currently under FDA review as a maintenance therapy in this subset of patients. Comprehensive genomic profiling has the potential to enable the identification of patients with specific alterations who may be candidates for immunotherapy and targeted therapies in the future. Finally, combination therapies that aim to reprogram the immunosuppressive tumor microenvironment in conjunction with immunotherapy are also being investigated, and have yielded some encouraging preliminary results (110).

## Conclusions

Thanks to the development of modern chemotherapeutic regimens, survival after surgery for PDAC has improved and pancreatologists worldwide believe that the treatment of PDAC demands a multidisciplinary approach. Today the role of NAT in PDAC is shifting from tumor shrinkage to controlling potential micrometastases and selecting patients who may benefit from radical resection. Given the absence of reliable predictors of resectability and some evidence supporting radical pancreatectomy with arterial resection, several papers advocate that all BR/LA patients with no progression on NAT should be offered surgical exploration. Increasing data demonstrating the benefits of NAT for BR/LA PDAC are driving a shift from up-front surgery to NAT in the multidisciplinary treatment of even potentially resectable PDAC. Although FOLFIRINOX is currently the most commonly used regimen in NAT, the jury is still out on the optimal approach in this setting—namely whether or not radiation therapy should be included, and if the chemotherapy should be FOLFIRINOX or gemcitabine-based. Several ongoing prospective trials will soon contribute further to our knowledge of the role of NAT for PDAC. Despite initial poor results with immunotherapy, comprehensive genomic profiling to identify cancers with specific deficits and combination therapies that aim to increase the immunogenicity of pancreas cancer may give immunotherapy a role in NAT for PDAC in the future.

## Author Contributions

AO, FH, QB, and MD: study conception and design and drafting of manuscript. AO, FH, and QB: acquisition of data. AO and FH: analysis and interpretation of data. MA-M, RS, and MD: critical revision. AO, FH, QB, MA-M, RS, and MD: final approval.

### Conflict of Interest

The authors declare that the research was conducted in the absence of any commercial or financial relationships that could be construed as a potential conflict of interest.
